# Heart rate variability is associated with left ventricular systolic, diastolic function and incident heart failure in the general population

**DOI:** 10.1186/s12916-022-02273-9

**Published:** 2022-02-21

**Authors:** Banafsheh Arshi, Sven Geurts, Martijn J. Tilly, Marten van den Berg, Jan A. Kors, Dimitris Rizopoulos, M. Arfan Ikram, Maryam Kavousi

**Affiliations:** 1 Department of Epidemiology, Erasmus MC - University Medical Center Rotterdam, Rotterdam, The Netherlands; 2 Department of Medical Informatics, Erasmus MC - University Medical Center Rotterdam, Rotterdam, The Netherlands

**Keywords:** Heart rate variability, Heart failure, Left ventricular systolic function, Left ventricular diastolic function

## Abstract

**Background:**

HRV has mostly shown associations with systolic dysfunction and more recently, with diastolic dysfunction in Heart failure (HF) patients. But the role of sympathetic nervous system in changes of left ventricular (LV) systolic and diastolic function and new-onset HF has not been extensively studied.

**Methods:**

Among 3157 men and 4405 women free of HF and atrial fibrillation retrospectively included from the population-based Rotterdam Study, we used linear mixed models to examine associations of RR-interval differences and standard deviation of RR-intervals corrected for heart rate (RMSSDc and SDNNc) with longitudinal changes of LV ejection fraction (LVEF), E/A ratio, left atrial (LA) diameter, E/e’ ratio. Afterwards, using cox regressions, we examined their association with new-onset HF.

**Results:**

Mean (SD) age was 65 (9.95) in men and 65.7 (10.2) in women. Every unit increase in log RMSSDc was accompanied by 0.75% (95%CI:-1.11%;-0.39%) and 0.31% (− 0.60%;-0.01%) lower LVEF among men and women each year, respectively. Higher log RMSSDc was linked to 0.03 (− 0.04;-0.01) and 0.02 (− 0.03;-0.003) lower E/A and also − 1.76 (− 2.77;− 0.75) and − 1.18 (− 1.99;-0.38) lower LVM index in both sexes and 0.72 mm (95% CI: − 1.20;-0.25) smaller LA diameters in women. The associations with LVEF in women diminished after excluding HF cases during the first 3 years of follow-up. During a median follow-up of 8.7 years, hazard ratios (95%CI) for incident HF were 1.34 (1.08;1.65) for log RMSSDc in men and 1.15 (0.93;1.42) in women. SDNNc showed similar associations.

**Conclusions:**

Indices of HRV were associated with worse systolic function in men but mainly with improvement in LA size in women. Higher HRV was associated with higher risk of new-onset HF in men. Our findings highlight potential sex differences in autonomic function underlying cardiac dysfunction and heart failure in the general population.

**Supplementary Information:**

The online version contains supplementary material available at 10.1186/s12916-022-02273-9.

## Background

Heart rate variability (HRV) is a standard non-invasive marker for evaluating the autonomic nervous system [1]. HRV is associated with diabetes, coronary atherosclerosis and adverse cardiac remodeling [2, 3]. Heart failure (HF), often characterized by signs of neurohumoral sympathetic activation, is considered a condition of autonomic imbalance.

Reduced HRV, has been shown in left ventricular (LV) systolic dysfunction [4–6]. HRV has mostly been associated with adverse outcomes in HF patients with reduced left ventricular ejection fraction (LVEF), establishing a link between autonomic derangement and systolic dysfunction. More recently, evidence on the role of increased sympathetic activity on the development of diastolic dysfunction in HF has emerged [7–9]. Inconclusive studies for therapies in patients with HF with preserved LVEF highlight the need for a better understanding of the pathophysiology of the sympathetic nervous system in LV function [7]. Despite few studies showing an association between HRV and incident HF in the elderly [2] or postmenopausal women [10], HRV before the onset of clinical symptoms of HF has not been extensively studied and data on the link between HRV and systolic and diastolic function prior to onset of HF are sparse. Parallel to sex differences in cardiac autonomic function and HRV, differences between women and men in cardiac function have also gained increasing attention as they play an important role in the pathophysiology of HF [11, 12]. Importantly, significant sex differences in the autonomic control of the heart mandate studies to avoid treating HRV equally in men and women [13].

Using serial measurements of systolic and diastolic cardiac function, we examined the association of ultra-short-term time-domain indices of HRV with evolution of cardiac function and with new onset HF among men and women from the large population-based Rotterdam Study.

## Methods

### Study population

**Fig. 1:**
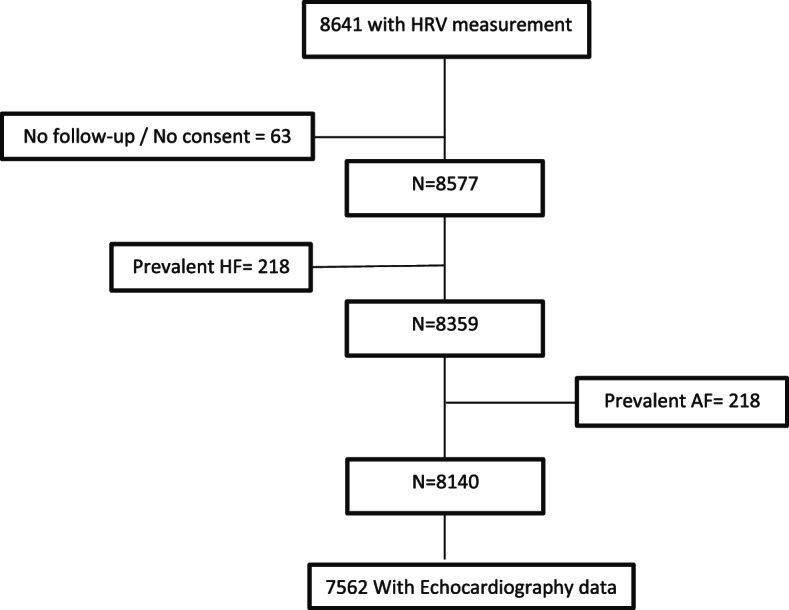
Flow chart of the study population

This project was conducted within the Rotterdam Study (RS), a prospective cohort study among individuals 45 ≥ years old living in Ommoord district of the city of Rotterdam, the Netherlands [14]. The first cohort of the Rotterdam Study (RS-I, 1990) included 7983 participants and was extended in 2000 (RS-II, *N* = 3011) and 2006 (RS-III, *N* = 3932). For this study, participants with HRV measurements at the fourth and fifth examinations of the first cohort (RS-I-4, RS-I-5), the second and third examinations of the second (RS-II-2, RS-II-3) and the first and second examinations of the third cohort (RS-III-1, R-III-2) were included (*N* = 8641). We excluded participants with a prior diagnosis of HF (*N* = 218), no follow-up data (*N* = 9), no consent for follow-up (*N* = 54) and prevalent atrial fibrillation (AF) (N = 218). Of these, 7562 had echocardiography measurements at the time of HRV measurement (Fig. 1).

The Rotterdam Study has been approved by the Medical Ethics Committee of Erasmus MC (registration number: MEC02.1015) and the Dutch Ministry of Health, Welfare and Sport (Population Screening Act WBO, license number:1071272–159,521-PG). The present study was performed in agreement with the principles outlined in the Declaration of Helsinki. All participants provided written informed consent for participation and having their information obtained from physicians during follow-up.

### Electrocardiogram interpretation and HRV measurement

Participants underwent a 10-s 12-lead resting electrocardiography using an ACTA Gnosis IV ECG recorder (Esaote Biomedica, Florence, Italy) with a sampling frequency of 500 Hz and results were digitally stored. Afterwards, measurements and interpretations were obtained by modular electrocardiographic analysis system (MEANS) which has been broadly appraised [15, 16]. For HRV, ECGs of individuals with pacemaker, those with less than 5 RR intervals, more than 5 premature ventricular and supraventricular complexes or with AF and flutter (manually confirmed by a cardiologist) were excluded [17]. Of the remaining ECGs, those marked as non-sinus arrhythmia and sinus arrhythmia were manually assessed by 2 medical doctors to rule out and exclude atrial fibrillation/flutter/other arrhythmias and ECGs with low quality/less than 5 consecutive RR intervals. Likewise, a sample of 200 ECGs marked as sinus rhythm were manually checked. RR intervals between two adjacent normal dominant beats were used to compute heart rate and time-domain indices of HRV; root mean square of successive RR-interval differences (RMSSD) and standard deviation of normal R-R intervals (SDNN). Due to the strong inverse, exponential association of HRV with heart rate, a linear adjustment with heart rate still leaves room for confounding by this factor [17, 18]. Therefore, we corrected RMSSD and SDNN using an exponential model [18]:

$$\mathrm{SDNNc}={\mathrm{SDNN}}^{\mathrm{e}\ast -0.02263\ \left(60-\mathrm{Heart}\ \mathrm{rate}\right)}$$

$$\mathrm{RMSSDc}={\mathrm{RMSSD}}^{\mathrm{e}\ast -0.03243\ \left(60-\mathrm{Heart}\ \mathrm{rate}\right)}$$

### Echocardiography

Participants went through resting transthoracic two-dimensional, M-mode and Doppler echocardiography by trained personnel [19], based on a standard protocol, using a commercially available ultrasonography system (AU3 Partner, Esaote Biomedica, with a 3.5/2.5 MHz transducer) until October 2003 and from then on by Acuson Cypress, Siemens, USA with a 3V2c transducer. From January 2009, a Vivid I (Vivid I, GE Healthcare, Little Chalfont, UK) with a 3S-RS Sector Array probe (1.5–3.6 MHz) was used. A maximum of three measurements were available for study participants. We used LVEF(%) calculated based on the Teichholz formula for LV systolic function, LV mass indexed by body surface area (LVM index) as an important predictor factor for HF [20], E wave/A wave ratio (E/A), left atrial anteroposterior diameter (LA diameter), E/e’ ratio (E/e’) for LV diastolic function. Detailed information is provided in Additional file 1: Methods S1 [19, 21].

### HF assessment

Prevalent and incident HF in the Rotterdam study are defined using clinical information from medical records according the European Society of Cardiology [22]. Follow-up information for HF was complete until January 2016. Detailed description is provided in Additional file 1: Methods S1 [22].

### Cardiovascular risk factors

Data collection for cardiovascular risk factors is described in Additional file 1: Methods S1 [14, 22, 23].

### Statistical analysis

Characteristics of study population were presented as mean (standard deviation [SD]) or median (interquartile range [IQR]) for continuous variables and numbers (%) for dichotomous variables. Variables were compared using Student T-test or Mann Whitney test based on their distribution. Due to their skewness, values of RMSSDc and SDNNc were natural log transformed to retain a normal distribution. Participants with complete data on covariates were included in the analyses (missing covariates< 4.5%).

To capture the association of baseline HRV measurements with cardiac function during follow-up, we used linear mixed effects models to account for the correlations in the repeated echocardiography measurements (a maximum of 3 measurements for LVEF, E/A, LA diameter and LVM index; 2 measurements for E/e’). For the random-effects, we used random intercepts and random slopes with an unstructured covariance matrix. Analyses were adjusted for age at each examination, hypertension, diabetes, coronary heart disease (CHD), total and HDL cholesterol, use of lipid lowering and cardiac medication and body mass index (BMI) (fixed-effect part). We also used a quadratic term for time of echocardiography and an interaction between indices of HRV and time of measurement. To check the non-linearity of HRV measurements, time of examination and interactions between covariates and time, we compared models with and without these terms using likelihood ratio test (*p*-values< 0.15 considered significant). Final models included a quadratic term for time for LVEF; for E/A, LA diameter and E/e’, a linear function of time was used.

Next, multivariable Cox proportional hazards regressions were used to estimate hazard ratios (HRs) of incident HF during follow-up in men and women. We used the counting process notation to account for entry time; event time was set as the time of incident HF or censoring. Furthermore, cause-specific hazards were assessed, with HF and mortality as competing events. Survival analyses were adjusted for hypertension, diabetes, CHD, total and HDL cholesterol, lipid lowering and cardiac medication and BMI. We also checked for nonlinearities in the associations of HRV with HF and interactions between indices of HRV with age, CHD, diabetes, hypertension, lipid lowering and cardiac medication (*p*-value< 0.15 considered significant).

To account for reverse causation in participants with cardiac dysfunction, we repeated our analyses excluding participants with incident HF within 3 years of follow-up. In ancillary analyses, longitudinal associations were performed using CHD events during follow-up as time-varying covariate and once replacing BMI with waist circumference. To compare our observations with the literature, we also performed the analyses using uncorrected indices of HRV, additionally adjusting for heart rate.

## Results

At baseline, mean (SD) age was 65 (9.95) in men and 65.7 (10.2) in women (Table 1). Diabetes and CHD were more frequent in men. While mean LVEF was higher in women, E/A was higher in men. Median (IQR) of RMSSDc was higher in women than men [32.30 (33.3) milliseconds in women vs 24.77 (29.3) milliseconds in men]; the same was observed for SDNNc [25.53 (25.8) milliseconds in women versus 21.91 (25.4) milliseconds in men].

**Table 1:** Baseline characteristics of the study population

	Men (3157)	Women (4405)	*p*-value
Age, years	65.0 (9.95)	65.7 (10.2)	0.005
Waist circumference, cm	98.83 (10.56)	89.21 (11.58)	< 0.001
BMI, Kg/m^2^	27.49 (3.64)	27.61 (4.65)	0.216
Total cholesterol, mg/dl	206.6 (38.6)	224.7 (39.0)	< 0.001
HDL, mg/dl	49.4 (13.1)	61.0 (16.2)	< 0.001
Diabetes, N(%)	422 (13.4)	427 (9.70)	< 0.001
CHD, N(%)	342 (10.9)	116 (2.70)	< 0.001
Hypertension, N(%)	2130 (67.8)	2859 (65.2)	0.021
Heart rate, beats/m	66.3 (10.5)	68.6 (9.63)	< 0.001
Cardiac medication, N(%)	174 (5.5)	198 (4.5)	0.050
Lipid lowering medication, N(%)	572 (26.1)	613 (20.6)	< 0.001
Smoking, N(%)			< 0.001
Current	736 (23.6)	777 (17.9)	–
Former	1837 (58.8)	1839 (42.3)	–
Never	552 (17.7)	1734 (39.9)	–
LVEF, %	64.29 (8.33)	66.28 (6.93)	< 0.001
LVEDD, mm	53.60 (4.90)	49.64 (4.51)	< 0.001
E/A ratio	0.97 (0.28)	0.94 (0.33)	0.001
LA diameter, mm	17.2 (7.78)	16.9 (6.68)	0.357
E/e’ ratio	9.48 (3.14)	10.5 (3.60)	< 0.001
LVM index, g/m^2^	52.4 (27.2)	55.7 (26.7)	< 0.001
RMSSDc, ms	24.77 (29.4)	32.30 (33.3)	< 0.001
SDNNc, ms	21.91 (25.4)	25.53 (25.8)	< 0.001
RR interval, ms	928.1 (145.2)	891.6 (125.3)	< 0.001

*BMI* Body mass index, *HDL* High density lipoprotein, *CHD* coronary heart disease, *LVEF* Left ventricular ejection fraction, *LVEDD* left ventricular end-diastolic diameter, *E/A* mitral E wave/ A wave ratio, *LA diameter* left atrial diameter, *E/e’* E wave/ septal e’ ratio, *LVM index* left ventricular mass index, *RMSSDc* root mean square of successive RR-interval differences corrected for heart rate, *SDNNc* standard deviation of normal R-R intervals corrected for heart rate

Missing values were present for total cholesterol (3%), HDL (3%), (4.7%), BMI (1.9%), waist circumference (1.6%), lipid-lowering medication (4.4%), smoking (1.1%), CHD (0.9%), cardiac medication (0.6%)

**Fig. 2:**
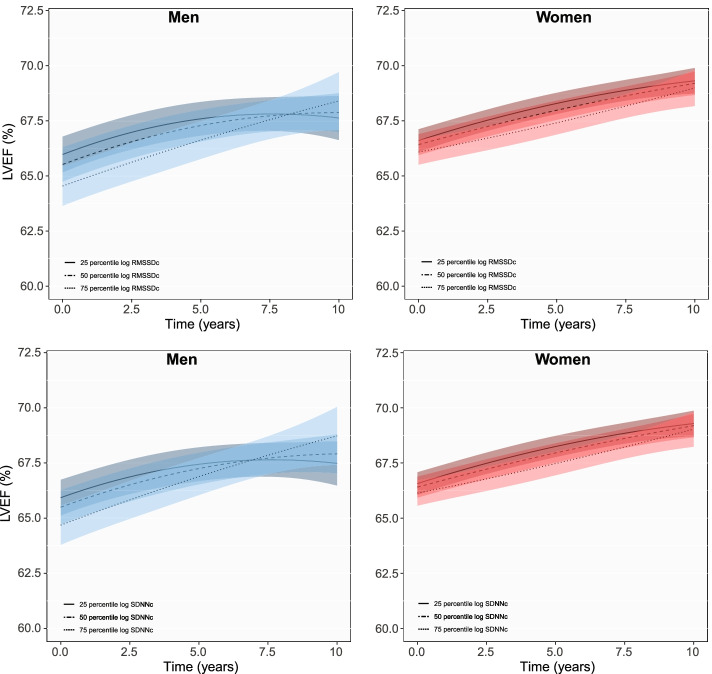
Longitudinal changes in LVEF with HRV in men and women. Figure shows changes in mean LVEF for the 25th, 50th and 75th percentile of each HRV measurement in men and women during follow-up

Every unit increase in the log RMSSDc was associated with 0.75% (95% CI: − 1.11% to − 0.39%) and 0.31% (− 0.60% to − 0.01%) lower LVEF among men and women at baseline, respectively (Table 2). Every unit increase in the log SDNNc was also linked to 0.72% (− 1.10% to − 0.34%) lower LVEF in men and 0.34% (− 0.52% to − 0.05%) lower LVEF in women. Figure 2 shows changes in mean LVEF for the 25th, 50th and 75th percentile of log RMSSDc and SDNNc during follow-up. Among men, the negative association persisted during follow-up, but those with higher RMSSDc and SDNNc showed a tendency towards higher LVEFs by the end of follow-up, although these changes were overlapping. Predicted LVEF with increasing RMSSDc and SDNNc in the 3rd, 6th and 9th year of follow-up are shown in Additional file 1: Fig. S1.

**Table 2:** Longitudinal associations of RMSSDc and SDNNc with echocardiographic parameters of left ventricular systolic and diastolic function in men and women

		RMSSDc^a^				SDNNc^a^			
		Men		Women		Men		Women	
		β (95% CI)	*p*-value	β (95% CI)	*p*-value	β (95% CI)	*p*-value	β (95% CI)	*p*-value
LVEF	HRV^b^	-0.75 (− 1.11 to − 0.39)	< 0.001	− 0.31 (− 0.60 to − 0.01)	0.042	− 0.72 (− 1.10 to − 0.34)	< 0.001	− 0.27 (− 0.59 to 0.05)	0.100
	HRV *time	− 0.02 (− 0.18 to 0.15)	0.831	− 0.10 (− 0.22 to 0.03)	0.145	0.02 (− 0.16 to 0.19)	0.870	−0.10 (− 0.23 to 0.04)	0.156
	HRV *time^2^	0.01 (−0.01 to 0.03)	0.189	0.01 (−0.004 to 0.03)	0.153	0.01 (−0.01 to 0.04)	0.246	0.01 (−0.004 to 0.03)	0.165
E/A	HRV	−0.03 (− 0.04 to − 0.01)	< 0.001	−0.02 (− 0.03 to − 0.003)	0.019	−0.02 (− 0.03 to − 0.01)	0.007	−0.01 (− 0.02 to 0.01)	0.399
	HRV *time	0.002 (0.001 to 0.01)	0.067	0.001 (−0.002 to 0.004)	0.473	0.001 (−0.001 to 0.01)	0.260	−0.001 (− 0.01 to 0.002)	0.707
LA diameter	HRV	0.23 (−0.26 to 0.73)	0.356	−0.72 (− 1.20 to − 0.25)	0.003	0.19 (− 0.34 to 0.72)	0.482	- 0.53 (− 1.02 to − 0.03)	0.037
	HRV *time	− 0.06 (− 0.14 to 0.02)	0.114	0.08 (0.01 to 0.16)	0.025	− 0.08 (− 0.17 to 0.002)	0.056	0.04 (− 0.03 to 0.12)	0.258
E/e’	HRV	− 0.09 (− 0.21 to 0.04)	0.165	0.01 (− 0.13 to 0.15)	0.890	− 0.11 (− 0.25 to 0.02)	0.101	0.03 (− 0.12 to 0.19)	0.678
	HRV *time	0.03 (− 0.03 to 0.09)	0.346	0.03 (− 0.04 to 0.10)	0.392	0.03 (− 0.04 to 0.09)	0.396	0.01 (− 0.06 to 0.08)	0.815
LVM index	HRV	− 1.76 (−2.77 to − 0.75)	0.001	− 1.18 (− 1.99 to − 0.38)	0.004	−1.83 (− 2.91 to − 0.76)	0.001	−1.35 (− 2.22 to − 0.49)	0.002
	HRV *time	0.0002 (− 0.21 to 0.21)	0.999	−0.09 (− 0.26 to 0.09)	0.327	0.004 (− 0.23 to 0.24)	0.970	− 0.15 (− 0.33 to 0.03)	0.111

^a^ Values of RMSSDc and SDNNc have been natural log transformed

^b^exposure (RMSSDc or SDNNc)

*RMSSDc* root mean square of successive RR-interval differences corrected for heart rate, *SDNNc* standard deviation of normal R-R intervals corrected for heart rate, *LVEF* Left ventricular ejection fraction, *E/A* mitral E wave/ A wave ratio, *LA diameter* left atrial diameter, *E/e’* E wave/ septal e’ ratio, *LVM index* left ventricular mass index. Analyses are adjusted for age, hypertension, diabetes, CHD, total cholesterol and HDL, use of lipid lowering and cardiac medication and BMI

**Fig. 3:**
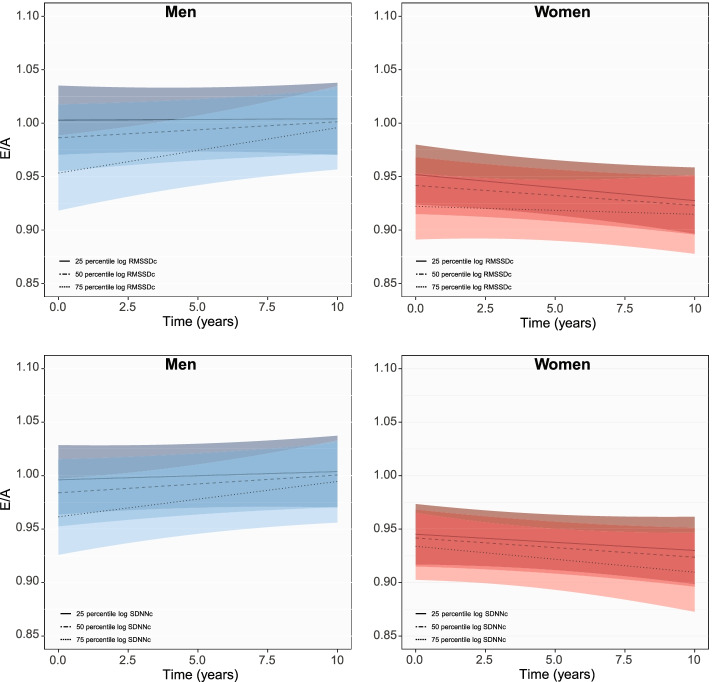
Longitudinal changes in E/A ratio with HRV in men and women Figure shows changes in mean E/A ratio for the 25th, 50th and 75th percentile of each HRV measurement in men and women during follow-up

At baseline, one unit higher log RMSSDc was associated with 0.03 (− 0.04 to − 0.01) and 0.02 (− 0.03 to − 0.003) lower mean E/A in men and women and SDNNc with 0.02 (− 0.03 to − 0.01) lower mean E/A in men (Table 2). Subtle changes towards weakening of these negative associations during follow-up (Fig. 3) were observed [time interaction (95% CI): 0.002 (0.001 to 0.01), Additional file 1: Fig. S2).

**Fig. 4:**
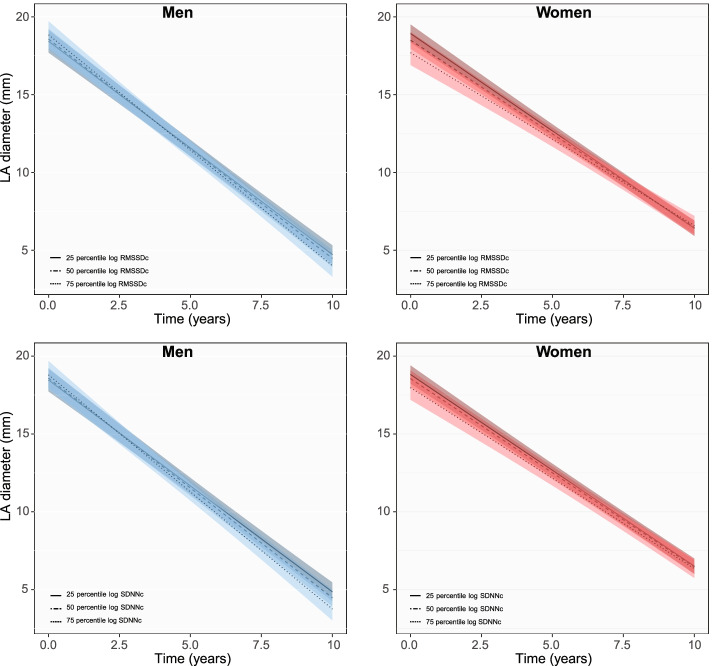
Longitudinal changes in LA diameter with HRV in men and women. Figure shows changes in mean LA diameter for the 25th, 50th and 75th percentile of each HRV measurement in men and women during follow-up

Increase in log RMSSDc and SDNNc at baseline were associated with 0.72 mm (95% CI: − 1.20 to − 0.25) and 0.53 mm (− 1.02 to − 0.03) smaller LA diameters in women, but not in men. Mean difference in LA size was gradually minimized until the end of follow-up (Fig. 4 for longitudinal changes, Additional file 1: Fig. S3 for the predictions at the 3rd, 6th and 9th year of follow-up).

**Fig. 5:**
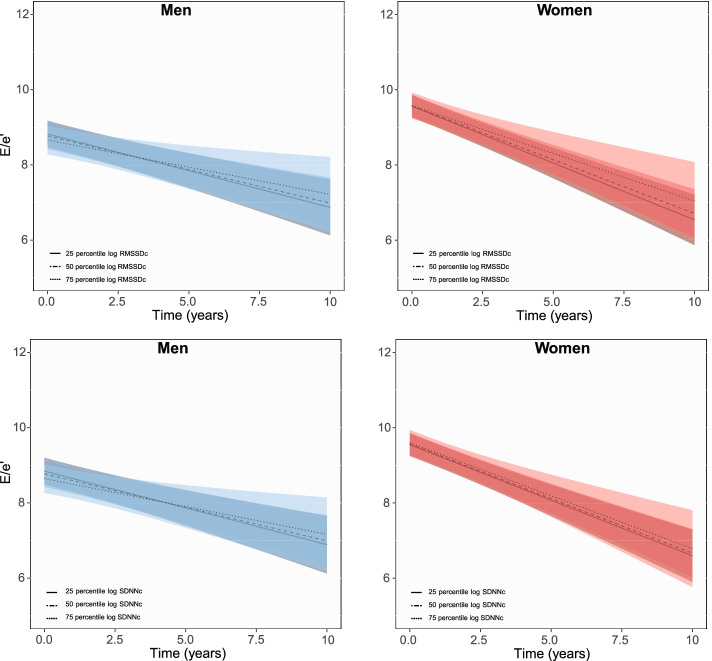
Longitudinal changes in E/e’ ratio with HRV in men and women. Figure shows changes in mean E/e’ ratio for the 25th, 50th and 75th percentile of each HRV measurement in men and women during follow-up

**Fig. 6:**
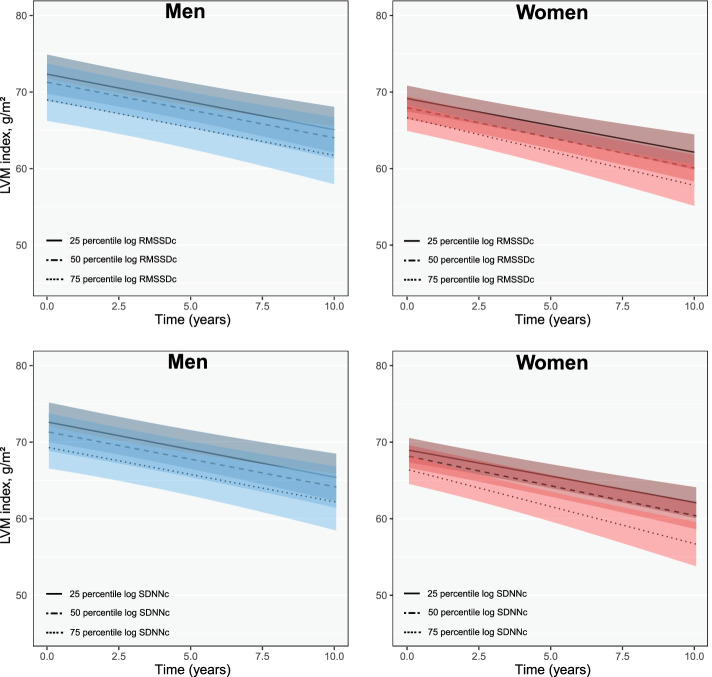
Longitudinal changes in LVM index with HRV in men and women. Figure shows changes in mean LVM index for the 25th, 50th and 75th percentile of each HRV measurement in men and women during follow-up

Men with higher log RMSSDc and SDNNc mainly had lower mean E/e’ [β (95% CI): − 0.09 (− 0.21 to 0.04) for RMSSDc and − 0.11 (− 0.25 to 0.02) for SDNNc] but women had higher mean E/e’s at baseline [β (95% CI): 0.01 (− 0.13 to 0.15) for RMSSDc and 0.03 (− 0.12 to 0.19) for SDNNc]; albeit non-significance (Fig. 5, Additional file 1: Fig. S4). LVM index was also lower with higher RMSSDc and SDNNc in men and women and these associations did not change drastically during follow-up (Fig. 6, Additional file 1: Fig. S5).

We observed significant interactions between HRV and LVEF for the associations with E/A and LA diameter in both sexes (all p-for interaction< 0.15). After stratification, increasing RMSSDc and SDNNc retained similar associations with E/A and LA diameter in those with LVEF> = 55 but not in those with LVEF< 55 (Additional file 1: Table. S1).

During a median follow-up of 8.7 years, 191 and 281 HF events were recorded in men and women, respectively (incidence rate: 7.46 and 7.47 per 1000 person-years in men and women). The HR (95% CI), every unit increase in log RMSSDc for incident HF was 1.34 (1.08 to 1.65) in men and 1.15 (0.93 to 1.42) in women (Table 3). Log SDNNc was also associated with higher HR for HF in men [1.30 (1.02 to 1.65)] but not women. Cause-specific HRs for incident HF with mortality as a competing event showed RMSSDc [1.38 (1.12 to 1.70)] and SDNNc [1.32 (1.03 to 1.68)] were associated with incident HF in men, but not in women.

**Table 3:** Hazard ratios (HRs) and 95% confidence intervals (95% CIs) of the association between RMSSDc and SDNNc with incident heart failure

	Men		Women	
	HR (95% CI)	*p*-value	HR (95% CI)	*p*-value
RMSSDc^a^	1.34 (1.08 to 1.65)	0.007	1.15 (0.93 to 1.42)	0.193
SDNNc^a^	1.30 (1.02 to 1.65)	0.033	1.10 (0.87 to 1.39)	0.438
Cause specific analysis				
RMSSDc^a^	1.38 (1.12 to 1.70)	0.003	1.17 (0.96 to 1.43)	0.112
SDNNc^a^	1.32 (1.03 to 1.68)	0.030	1.13 (0.91 to 1.41)	0.268

^a^ Values of RMSSDc and SDNNc have been natural log transformed

*RMSSDc* root mean square of successive RR-interval differences corrected for heart rate, *SDNNc* standard deviation of normal R-R intervals corrected for heart rate. Analyses are adjusted for age, hypertension, diabetes, CHD, total cholesterol and HDL, use of lipid lowering and cardiac medication, BMI

Excluding participants with incident HF during the first 3 years of follow-up did not change our results (Additional file 1: Table. S2 and Table. S3). Sensitivity analyses using CHD as a time dependent covariate and waist circumference instead of BMI in the analyses yielded similar results (data not shown). Finally, using uncorrected indices of HRV while adjusting for heart rate, the associations of RMSSD and SDNN with LVEF were attenuated. This is while RMSSD and SDNN associated with higher E/A, opposite to RMSSDc and SDNNc (Additional file 1: Table. S4). Lastly, higher RMSSD and SDNN showed smaller increase in the risk of HF (Additional file 1: Table. S5).

## Discussion

Individuals with higher ultra-short-term time-domain indices of HRV had worse LV systolic function in men and women, particularly men. There were small changes in the longitudinal association and there was a tendency for higher LVEF with higher HRV towards the end of follow-up in men. Higher HRV, specially RMSSDc, was linked to lower E/A in both sexes; these associations did not largely change with time. Women with higher HRV, but not men, had smaller LVM index and LA size; the difference in LA diameter for higher HRV grew smaller during follow-up. Finally, higher HRV was associated with higher risk of incident HF, specifically in men.

A deranged autonomic nervous system might be associated with cardiac dysfunction and incident HF [2]. Autonomic imbalance can also be linked to HF through cardiovascular risk factors including obesity, diabetes and CHD which play a role in the pathophysiology of HF [2, 4, 17, 24]. It has also been postulated that HRV is associated with inflammation, a central pathophysiological mechanism in the development of HF [3]. HRV has mostly been associated with adverse outcomes in patients with reduced LVEF, establishing a link between autonomic derangement and systolic dysfunction. More recently, evidence on the role of increased sympathetic activity on the development of diastolic dysfunction in HF has emerged [7–9]. Increase in collagen and elastic fibers with aging can result in pathologic changes in the sinoatrial node, resulting in sinus node dysfunction [25, 26]. Collagen over-expression is linked to diastolic dysfunction in HF with preserved LVEF [7]. But these associations have not been extensively studied in the general population before.

Investigating sex-specific mechanisms in HF provides insights for more efficient strategies for disease management [11]. Higher HRV, especially RMSSDc, was associated with higher risk of new-onset HF in men than in women in our study. This could be attributed to the overall greater impact of HRV on unfavorable systolic function in men, also attributable to male predisposition to HF with reduced LVEF [11]. In our study, the inverse association weakened towards later years of follow-up in men which could due to higher mortality rate among men with lower LVEF and survival of those with higher LVEF at later years. Among women, the observed risk of HF with increasing HRV was smaller and insignificant which could be explained by the weaker association of HRV with LVEF in women and also its favorable link with parameters of diastolic function. Exclusion of women with incident HF early during follow-up was followed by further weakening of the association between HRV with LVEF but no drastic change in the association with LA size and conceivably, no increased risk of HF. Our study provides further evidence supporting the role of the sympathetic nervous system in diastolic function, especially in women [7].

We consistently observed similar associations between HRV and diastolic function among individuals with LVEF> = 50 but not in those with LVEF< 50 in our sensitivity analyses. Due to possible variabilities in sympathetic nerve activity in HF with preserved and reduced LVEF, investigation of these differences could help with developing better approaches for HF treatment [7]. A cross-sectional study among HF patients has previously shown decreased HRV in HF with both reduced and preserved LVEF [9]. Our results suggest the possibility of a stronger link between the sympathetic nerve activity and favorable diastolic function in asymptomatic individuals with preserved LVEF. Diastolic dysfunction, as a hallmark of HF with preserved LVEF, is more frequently observed in women than men [11]. And, LA dysfunction plays a major role in the pathophysiology of HF with preserved LVEF [11]. This could also explain the more prominent associations found between HRV and parameters of diastolic function in women. However, fewer number of individuals with lower LVEF could have resulted in the insignificant associations in our study.

Our findings regarding the favorable link between higher HRV and diastolic function are in line with previous studies [27]. However, associations of higher HRV with pathological conditions are less reported. Although decreased HRV has usually been considered pathological, contradictory reports also exist [1]. We report increased risk of incident HF, coupled with a more prominent association of higher HRV with worsening of systolic function, specifically in men. Comparing our findings with other investigations, several factors need to be accounted for. With time domain parameters of HRV, only studies with the same length of recording can be compared [28]. Another study, based on 10-s ECGs, has reported higher risk of incident HF with high HRV (RMSSD> 44 ms) [10]. Also, higher HRV has shown to be a stronger indicator of cardiac mortality than lower HRV in the Rotterdam study [25]. Decreased HRV has been mainly reported in HF patients but there are also reports contradicting the assumption that HF is accompanied by a high sympathetic tone [4]. Thus, interpretation and generalization of HRV analyses in certain groups and among HF patients and individuals free of HF from the general population may not be straightforward [4]. In addition, HRV has a strong inverse exponential association with HR and concurrently, high resting HR (lower HRV) is associated with poor cardiovascular profiles [18, 29, 30]. While we used HR-corrected measurements, other studies have mostly adjusted their analysis with HR, leaving room for further confounding by this factor [18]. However, when we used uncorrected HRV, some associations with cardiac function changed but the overall link to incident HF did not alter. Another important reason for our differences could be the procedures to certify HRV measurements [25]. To note, increased HRV is accompanied by irregular sinus arrhythmia which cannot be extinguished from normal sinus arrhythmia with conventional measures and could result in the observed higher risk with increasing HRV [25]. Finally, differences in age and variabilities in the adjudication of HF and covariates between studies merit attention.

Strengths of our study are its large sample from a population-based cohort, well-defined covariates and well adjudicated HF events. We also benefited from a detailed screening of ECGs. Also, a standard protocol was used by trained echocardiographers with good inter-reader and intra-reader agreement to minimize limitations of echocardiography as an operator-dependent tool [31, 32]. Moreover, longitudinal analysis of outcomes using robust statistical models further support our findings. There are also limitations. LVEF was calculated by linear end-systolic and diastolic LV dimensions using the Teichholz formula which can overestimate LVEF in case of wall motion abnormalities [21]. Second, measurement of biplane LA volume and lateral e’ were not performed in the examinations and limit an ideal assessment of LV diastolic function in our study. Third, compared to the gold-standard 24 h recordings, 10-s ECGs limited our ability to take fluctuations of this measurement into account [28]. RMSSD is used to estimate the vagal changes reflected in HRV while SDNN reflects autonomic influence on HRV and correlates with very low frequency power. Thus, the direct association of HRV frequency components with divisions of the autonomic nervous system could be too simplistic. Additional assessment of peak heart rate during exercise test in patients without overt HF also has a key clinical meaning and can reflect the dynamic balance between parasympathetic and sympathetic drives [33]. Despite adjusting for possible confounders, residual confounding cannot be ruled out. Finally, population were mainly older individuals of European decent, necessitating caution in generalizing our observations.

## Conclusions

We show that autonomic imbalance may be involved in the pathogenesis of HF through changes in cardiac function, independent of other cardiovascular risk factors. Increase in indices of HRV was mainly associated with greater decline in LV systolic function in men than in women. The association between Increasing HRV and diastolic function was smaller but favorable, especially for LA size in women. Increasing HRV, was associated with higher risk of new-onset HF in men.

## Supplementary Information

**Additional file 1 Table S1.** Longitudinal association of RMSSDc and SDNNc with echocardiographic parameters of left ventricular systolic and diastolic function in men and women, by LVEF category. **Table S2.** Longitudinal association of RMSSDc and SDNNc with echocardiographic parameters of left ventricular systolic and diastolic function in men and women. **Table S3.** Hazard ratios (HRs) and 95% confidence intervals (95% CIs) of the association between RMSSDc. and SDNNc with incident heart failure. **Table S4.** Longitudinal associations of RMSSD and SDNN with echocardiographic parameters of left ventricular systolic and diastolic function in men and women. **Table S5.** Hazard ratios (HRs) and 95% confidence intervals (95% CIs) of the association between RMSSD and SDNN with incident heart failure. **FigS1.** Association of RMSSDc and SDNNc with LVEF at year 3, 6 and 9 of follow-up. **FigS2.** Association of RMSSDc and SDNNc with E/A ratio at year 3, 6 and 9 of follow-up. **FigS3.** Association of RMSSDc and SDNNc with LA diameter at year 3, 6 and 9 of follow-up. **FigS4**. Association of RMSSDc and SDNNc with E/e’ ratio at year 3, 6 and 9 of follow-up. **FigS5**. Association of RMSSDc and SDNNc with LVM index at year 3, 6 and 9 of follow-up. **Methods S1.** Details on methods of data collection in the study.

## Data Availability

The analyzed datasets during the current study are not publicly available due to legal and ethical restraints. Sharing of individual participant data was not included in the informed consent of the study, and there is potential risk of revealing participants’ identities as it is not possible to completely anonymize the data. However, data are available from the corresponding author on reasonable request.

## Abbreviations

HRV: Heart rate variability
HF: Heart failure
LV systolic dysfunction: Left ventricular systolic dysfunction
LVEF: Left ventricular ejection fraction
MEANS: Modular electrocardiographic analysis system
RMSSD: RR-interval differences of normal R-R intervals
SDNN: Standard deviation of normal R-R intervals
RMSSDc: RR-interval differences of normal R-R intervals corrected for heart rate
SDNNc: Standard deviation of normal R-R intervals corrected for heart rate
CHD: Coronary heart disease
BMI: Body mass index
LVM index: LV mass indexed by body surface area
E/A: E wave/A wave ratio
LA diameter: Left atrial anteroposterior diameter
E/e’: E/e’ ratio
SD: Standard deviation
IQR: Interquartile range
