# Supplementary material for: Heart rate variability is associated with left ventricular systolic, diastolic function and incident heart failure in the general population
Source: BMC Med. 2022 Feb 21;20:91. doi: 10.1186/s12916-022-02273-9 (PMC8862241; doi:10.1186/s12916-022-02273-9)
Supplement: Supplementary file 1 — Additional file 1 Table S1. Longitudinal association of RMSSDc and SDNNc with echocardiographic parameters of left ventricular systolic and diastolic function in men and women, by LVEF category. Table S2. Longitudinal association of RMSSDc and SDNNc with echocardiographic parameters of left ventricular systolic and diastolic function in men and women. Table S3. Hazard ratios (HRs) and 95% confidence intervals (95% CIs) of the association between RMSSDc. and SDNNc with incident heart failure. Table S4. Longitudinal associations of RMSSD and SDNN with echocardiographic parameters of left ventricular systolic and diastolic function in men and women. Table S5. Hazard ratios (HRs) and 95% confidence intervals (95% CIs) of the association between RMSSD and SDNN with incident heart failure. FigS1. Association of RMSSDc and SDNNc with LVEF at year 3, 6 and 9 of follow-up. FigS2. Association of RMSSDc and SDNNc with E/A ratio at year 3, 6 and 9 of follow-up. FigS3. Association of RMSSDc and SDNNc with LA diameter at year 3, 6 and 9 of follow-up. FigS4. Association of RMSSDc and SDNNc with E/e’ ratio at year 3, 6 and 9 of follow-up. FigS5. Association of RMSSDc and SDNNc with LVM index at year 3, 6 and 9 of follow-up. Methods S1. Details on methods of data collection in the study. [file 12916_2022_2273_MOESM1_ESM.docx]

**Additional Data**

|  | **Page** |
| --- | --- |
| **Table S1.** Longitudinal association of RMSSDc and SDNNc with echocardiographic parameters of left ventricular systolic and diastolic function in men and women, by LVEF category | 2-3 |
| **Table S2.** Longitudinal association of RMSSDc and SDNNc with echocardiographic parameters of left ventricular systolic and diastolic function in men and women^a^ | 5 |
| **Table S3.** Hazard ratios (HRs) and 95% confidence intervals (95% CIs) of the association between RMSSDc  and SDNNc with incident heart failure^a^ | 6 |
| **Table S4.** Longitudinal associations of RMSSD and SDNN with echocardiographic parameters of left ventricular systolic and diastolic function in men and women | 7 |
| **Table S5.** Hazard ratios (HRs) and 95% confidence intervals (95% CIs) of the association between RMSSD and SDNN with incident heart failure | 8 |
| **Figure S1.** Association of RMSSDc and SDNNc with LVEF at year 3, 6 and 9 of follow-up | 9 |
| **Figure S2.** Association of RMSSDc and SDNNc with E/A ratio at year 3, 6 and 9 of follow-up | 10 |
| **Figure S3.** Association of RMSSDc and SDNNc with LA diameter at year 3, 6 and 9 of follow-up | 11 |
| **Figure S4.** Association of RMSSDc and SDNNc with E/e’ ratio at year 3, 6 and 9 of follow-up | 12 |
| **Figure S5.** Association of RMSSDc and SDNNc with LVM index at year 3, 6 and 9 of follow-up | 13 |
| **Methods S1.** Details on methods of data collection in the study | 14-15 |

**Table S1.** Longitudinal association of RMSSDc and SDNNc with echocardiographic parameters of left ventricular systolic and diastolic function in men and women, by LVEF category

|  |  | RMSSDc^a^ | | | | |  | SDNNc^a^ | | | | |
| --- | --- | --- | --- | --- | --- | --- | --- | --- | --- | --- | --- | --- |
|  |  | Men | |  | Women | |  | Men | |  | Women | |
|  |  | β (95% CI) | p-value |  | β (95% CI) | p-value |  | β (95% CI) | p-value |  | β (95% CI) | p-value |
| LVEF >=50 | | | | | | | | | | | | |
| E/A | HRV^b^ | -0.03 (-0.04 to -0.01) | 0.000 |  | -0.02 (-0.03 to -0.01) | 0.004 |  | -0.02 (-0.03 to -0.004) | 0.014 |  | -0.02 (-0.03 to -0.002) | 0.020 |
|  | HRV *time | 0.002 (-0.0002 to 0.01) | 0.067 |  | 0.001 (-0.002 to 0.003) | 0.498 |  | 0.001 (-0.001 to 0.004) | 0.267 |  | -0.001 (-0.004 to 0.002) | 0.488 |
| LA diameter | HRV | 0.14 (-0.39 to 0.67) | 0.609 |  | -0.75 (-1.23 to -0.27) | 0.002 |  | 0.15 (-0.42 to 0.72) | 0.605 |  | -0.54 (-1.04 to -0.04) | 0.035 |
|  | HRV *time | -0.05 (-0.13 to 0.04) | 0.277 |  | 0.09 (0.02 to 0.16) | 0.019 |  | -0.07 (-0.16 to 0.02) | 0.115 |  | 0.05 (-0.03 to 0.12) | 0.219 |
| E/e’ | HRV | -0.01 (-0.23 to -0.01) | 0.032 |  | 0.02 (-0.13 to 0.13) | 0.830 |  | -0.12 (-0.26 to -0.001) | 0.037 |  | 0.01 (-0.12 to 0.21) | 0.578 |
|  | HRV *time | 0.04 (-0.02 to 0.097) | 0.236 |  | 0.01 (-0.06 to 0.06) | 0.524 |  | 0.03 (-0.03 to 0.10) | 0.351 |  | -0.01 (-0.08 to 0.06) | 0.660 |
| LVM index | HRV | -1.48 (-2.48 to -0.47) | 0.004 |  | -1.24 (-2.03 to -0.45) | 0.002 |  | -1.70 (-2.77 to -0.64) | 0.002 |  | -1.30 (-2.15 to -0.44) | 0.003 |
|  | HRV *time | 0.05 (-0.16 to 0.25) | 0.648 |  | -0.07 (-0.25 to 0.10) | 0.419 |  | 0.08 (-0.15 to 0.30) | 0.504 |  | -0.15 (-0.33 to 0.04) | 0.118 |
| LVEF<50 | | | | | | | | | | | | |
| E/A | HRV | -0.06 (-0.13 to 0.02) | 0.158 |  | 0.08 (-0.45 to 0.61) | 0.774 |  | -0.04 (-0.12 to 0.04) | 0.328 |  | 0.12 (-0.43 to 0.68) | 0.655 |
|  | HRV *time | 0.02 (-0.06 to 0.10) | 0.622 |  | -0.02 (-0.07 to 0.03) | 0.229 |  | -0.004 (-0.03 to 0.02) | 0.704 |  | -0.03 (-0.07 to 0.02) | 0.147 |
| LA diameter | HRV | -1.25 (-5.39 to 2.90) | 0.543 |  | -0.43 (-3.11 to 2.25) | 0.746 |  | -1.39 (-6.07 to 3.29) | 0.550 |  | -0.83 (-3.72 to 2.07) | 0.568 |
|  | HRV *time | -0.08 (-0.77 to 0.61) | 0.789 |  | -0.05 (-5.92 to 6.01) | 0.669 |  | -0.11 (-0.91 to 0.70) | 0.754 |  | -0.08 (-9.50 to 4.32) | 0.525 |
| E/e’ | HRV | 0.94 (-0.63 to 2.50) | 0.234 |  | -0.99 (-2.68 to 0.69) | 0.222 |  | 0.65 (-0.99 to 2.29) | 0.425 |  | -0.24 (-2.76 to 2.29) | 0.850 |
|  | HRV *time | -0.11 (-1.24 to 1.02) | 0.804 |  | 0.21 (-2.05 to 3.05) | 0.121 |  | 0.09 (-0.93 to 1.11) | 0.816 |  | 0.99 (-3.66 to 4.51) | 0.447 |
| LVM index | HRV | -9.42 (-16.7 to -2.10) | 0.012 |  | -2.07 (-11.6 to 7.48) | 0.663 |  | -6.26 (-14.0 to 1.52) | 0.113 |  | -4.46 (-14.5 to 5.63) | 0.377 |
|  | HRV *time | -1.62 (-3.61 to 0.36) | 0.097 |  | -0.21 (-23.6 to 23.1) | 0.927 |  | -1.88 (-3.95 to 0.19) | 0.070 |  | 0.06 (-26.9 to 27.1) | 0.981 |

^a^ Values of RMSSDc and SDNNc have been natural log transformed.

RMSSDc: root mean square of successive RR-interval differences corrected for heart rate, SDNNc: standard deviation of normal R-R intervals corrected for heart rate, LVEF: Left ventricular ejection fraction, LVEDD: left ventricular end-diastolic dimension, E/A; mitral E wave/ A wave ratio, LA diameter: left atrial diameter, E/e’: E wave/ septal e’ ratio, LVM index; left ventricular mass index. Analyses are adjusted for age, hypertension, diabetes, CHD, total cholesterol and HDL, use of lipid lowering and cardiac medication, BM

**Table S2.** Longitudinal association of RMSSDc and SDNNc with echocardiographic parameters of left ventricular systolic and diastolic function in men and women^a^

|  |  | RMSSDc^b^ | | | | |  | SDNNc^b^ | | | | |
| --- | --- | --- | --- | --- | --- | --- | --- | --- | --- | --- | --- | --- |
|  |  | Men | |  | Women | |  | Men | |  | Women | |
|  |  | β (95% CI) | p-value |  | β (95% CI) | p-value |  | β (95% CI) | p-value |  | β (95% CI) | p-value |
| LVEF | HRV^c^ | -0.65 (-1.01 to -0.3) | 0.000 |  | -0.21 (-0.50 to 0.09) | 0.168 |  | -0.64 (-1.02 to -0.26) | 0.001 |  | -0.16 (-0.48 to 0.15) | 0.311 |
|  | HRV:time | -0.05 (-0.22 to 0.11) | 0.537 |  | -0.12 (-0.25 to 0.01) | 0.072 |  | -0.01 (-0.19 to 0.16) | 0.882 |  | -0.13 (-0.26 to 0.01) | 0.064 |
|  | HRV:time^2^ | 0.02 (-0.01 to 0.04) | 0.123 |  | 0.01 (-0.003 to 0.03) | 0.113 |  | 0.02 (-0.01 to 0.04) | 0.183 |  | 0.01 (-0.003 to 0.03) | 0.103 |
| E/A | HRV | -0.03 (-0.04 to -0.02) | 0.000 |  | -0.02 (-0.03 to -0.002) | 0.027 |  | -0.02 (-0.04 to -0.01) | 0.003 |  | -0.01 (-0.02 to 0.01) | 0.481 |
|  | HRV:time | 0.002 (0.0001 to 0.01) | 0.060 |  | 0.001 (-0.002 to 0.004) | 0.549 |  | 0.001 (-0.001 to 0.01) | 0.266 |  | -0.001 (-0.01 to 0.002) | 0.639 |
| LA dimension | HRV | 0.21 (-0.29 to 0.70) | 0.415 |  | -0.71 (-1.18 to -0.23) | 0.004 |  | 0.18 (-0.35 to 0.71) | 0.513 |  | -0.5 (-1.00 to -0.01) | 0.048 |
|  | HRV:time | -0.06 (-0.14 to 0.02) | 0.142 |  | 0.08 (0.01 to 0.15) | 0.027 |  | -0.08 (-0.17 to 0.004) | 0.062 |  | 0.04 (-0.03 to 0.12) | 0.272 |
| E/e’ | HRV | -0.09 (-0.22 to 0.03) | 0.147 |  | 0.01 (-0.13 to 0.15) | 0.892 |  | -0.12 (-0.25 to 0.02) | 0.089 |  | 0.04 (-0.12 to 0.19) | 0.644 |
|  | HRV:time | 0.03 (-0.03 to 0.09) | 0.356 |  | 0.03 (-0.04 to 0.09) | 0.393 |  | 0.03 (-0.04 to 0.09) | 0.398 |  | 0.01 (-0.06 to 0.08) | 0.722 |
| LVM index | HRV | -1.97 (-2.98 to -0.96) | 0.000 |  | -1.11 (-1.91 to -0.30) | 0.007 |  | -1.98 (-3.06 to -0.91) | 0.000 |  | -1.28 (-2.15 to -0.41) | 0.004 |
|  | HRV:time | 0.01 (-0.20 to 0.23) | 0.897 |  | -0.10 (-0.28 to 0.08) | 0.277 |  | 0.02 (-0.22 to 0.25) | 0.898 |  | -0.16 (-0.34 to 0.03) | 0.098 |

RMSSDc: root mean square of successive RR-interval differences corrected for heart rate, SDNNc: standard deviation of normal R-R intervals corrected for heart rate, LVEF: Left ventricular ejection fraction, E/e’: E wave/ septal e’ ratio , LVM index; left ventricular mass index. Analyses are adjusted for age, hypertension, diabetes, CHD, total cholesterol and HDL, use of lipid lowering and cardiac medication and BMI.

^a^ Analyses were performed after excluding 60 men and 57 women with incident HF less than three years after entering the study

^b^ Values of RMSSDc and SDNNc have been natural log transformed.

^c^ RMSSDc or SDNNc

**Table S3.** Hazard ratios (HRs) and 95% confidence intervals (95% CIs) of the association between RMSSDc and SDNNc with incident heart failure^a^

|  |  | Men | |  | Women | |
| --- | --- | --- | --- | --- | --- | --- |
|  |  | HR (95% CI) | p-value |  | HR (95% CI) | p-value |
| RMSSDc^b^ |  | 1.31 (1.03 to 1.66) | 0.026 |  | 1.04 (0.83 to 1.29) | 0.750 |
| SDNNc^b^ |  | 1.25 (0.94 to 1.66) | 0.120 |  | 0.98 (0.76 to 1.25) | 0.846 |
| Cause specific analysis | | | | | | |
| RMSSDc |  | 1.33 (1.06 to 1.67) | 0.015 |  | 1.08 (0.88 to 1.33) | 0.452 |
| SDNNc |  | 1.25 (0.94 to 1.67) | 0.121 |  | 1.03 (0.82 to 1.3) | 0.783 |

RMSSDc: root mean square of successive RR-interval differences corrected for heart rate, SDNNc: standard deviation of normal R-R intervals corrected for heart rate. Analyses are adjusted for age, hypertension, diabetes, CHD, total cholesterol and HDL, use of lipid lowering and cardiac medication and BMI.

^a^ Analyses were performed after excluding 60 men and 57 women with incident HF less than three years after entering the study b Values of RMSSDc and SDNNc have been natural log transformed.

^b^ Values of RMSSDc and SDNNc have been natural log transformed.

**Table S4.** Longitudinal associations of RMSSD and SDNN with echocardiographic parameters of left ventricular systolic and diastolic function in men and women

|  |  | RMSSD^a^ | | | | |  | SDNN^a^ | | | | |
| --- | --- | --- | --- | --- | --- | --- | --- | --- | --- | --- | --- | --- |
|  |  | Men | |  | Women | |  | Men | |  | Women | |
|  |  | β (95% CI) | p-value |  | β (95% CI) | p-value |  | β (95% CI) | p-value |  | β (95% CI) | p-value |
| LVEF | HRV^c^ | -0.40 (-0.82 to 0.02) | 0.062 |  | -0.004 (-0.34 to 0.34) | 0.979 |  | -0.41 (-0.83 to 0.01) | 0.054 |  | -0.01 (-0.35 to 0.33) | 0.951 |
|  | HRV:time | -0.01 (-0.20 to 0.17) | 0.888 |  | -0.10 (-0.20 to 0.07) | 0.344 |  | 0.04 (-0.15 to 0.22) | 0.685 |  | -0.07 (-0.21 to 0.06) | 0.292 |
|  | HRV:time^2^ | 0.01 (-0.01 to 0.04) | 0.239 |  | 0.002 (-0.01 to 0.02) | 0.748 |  | 0.01 (-0.01 to 0.03) | 0.386 |  | 0.01 (-0.01 to 0.02) | 0.564 |
| E/A | HRV | 0.04 (0.02 to 0.05) | 0.000 |  | 0.03 (0.02 to 0.05) | 0.000 |  | 0.03 (0.01 to 0.04) | 0.000 |  | 0.03 (0.01 to 0.05) | 0.000 |
|  | HRV:time | -0.004 (-0.01 to -0.001) | 0.003 |  | -0.002 (-0.01 to 0.001) | 0.116 |  | -0.003 (-0.01 to -0.001) | 0.014 |  | -0.003 (-0.01 to 0) | 0.029 |
| LA diameter | HRV | 0.47 (-0.08 to 1.02) | 0.093 |  | -0.44 (-0.95 to 0.08) | 0.099 |  | 0.30 (-0.25 to 0.84) | 0.292 |  | -0.30 (-0.8 to 0.23) | 0.266 |
|  | HRV:time | -0.08 (-0.17 to 0.004) | 0.062 |  | 0.05 (-0.03 to 0.12) | 0.219 |  | -0.09 (-0.18 to -0.003) | 0.042 |  | 0.02 (-0.06 to 0.09) | 0.699 |
| E/e | HRV | -0.002 (-0.14 to 0.14) | 0.976 |  | 0.08 (-0.09 to 0.24) | 0.359 |  | -0.05 (-0.20 to 0.10) | 0.504 |  | 0.08 (-0.08 to 0.25) | 0.323 |
|  | HRV:time | 0.02 (-0.05 to 0.09) | 0.561 |  | 0.04 (-0.03 to 0.12) | 0.280 |  | 0.02 (-0.05 to 0.09) | 0.565 |  | 0.01 (-0.06 to 0.09) | 0.741 |
| LVM index | HRV | 1.69 (0.57 to 2.80) | 0.003 |  | 0.77 (-0.10 to 1.64) | 0.081 |  | 0.74 (-0.41 to 1.89) | 0.205 |  | 0.17 (-0.74 to 1.08) | 0.718 |
|  | HRV:time | 0.09 (-0.15 to 0.33) | 0.454 |  | -0.08 (-0.26 to 0.10) | 0.392 |  | 0.075 (-0.17 to 0.32) | 0.545 |  | -0.15 (-0.33 to 0.04) | 0.126 |

RMSSD: root mean square of successive RR-interval differences, SDNN: standard deviation of normal R-R intervals, LVEF: Left ventricular ejection fraction, E/A; mitral E wave/ A wave ratio, LA diameter: left atrial diameter, E/e’: E wave/ septal e’ ratio, LVM index; left ventricular mass index. Analyses are adjusted for age, heart rate, hypertension, diabetes, CHD, total cholesterol and HDL, use of lipid lowering and cardiac medication, BMI.

a Values of RMSSD and SDNN have been natural log transformed.

**Table S5.** Hazard ratios (HRs) and 95% confidence intervals (95% CIs) of the association between RMSSD and SDNN with incident heart failure

|  |  | Men | |  | Women | |
| --- | --- | --- | --- | --- | --- | --- |
|  |  | HR (95% CI) | p-value |  | HR (95% CI) | p-value |
| RMSSD^a^ |  | 1.32 (1.05 to 1.65) | 0.019 |  | 1.04 (0.82 to 1.34) | 0.729 |
| SDNN^a^ |  | 1.26 (0.97 to 1.62) | 0.078 |  | 1.00 (0.77 to 1.3) | 0.995 |
| Cause specific analysis | | | | | | |
| RMSSD |  | 1.31 (1.06 to 1.63) | 0.014 |  | 1.04 (0.83 to 1.3) | 0.711 |
| SDNN |  | 1.27 (0.99 to 1.64) | 0.063 |  | 1.02 (0.8 to 1.3) | 0.857 |

RMSSDc: root mean square of successive RR-interval differences, SDNNc: standard deviation of normal R-R intervals. Analyses were adjusted for age, heart rate, hypertension, diabetes, CHD, total cholesterol and HDL, use of lipid lowering and cardiac medication and BMI.

a Values of RMSSD and SDNN have been natural log transformed.

**Figure S1.** Association of RMSSDc and SDNNc with LVEF at year 3, 6 and 9 of follow-up


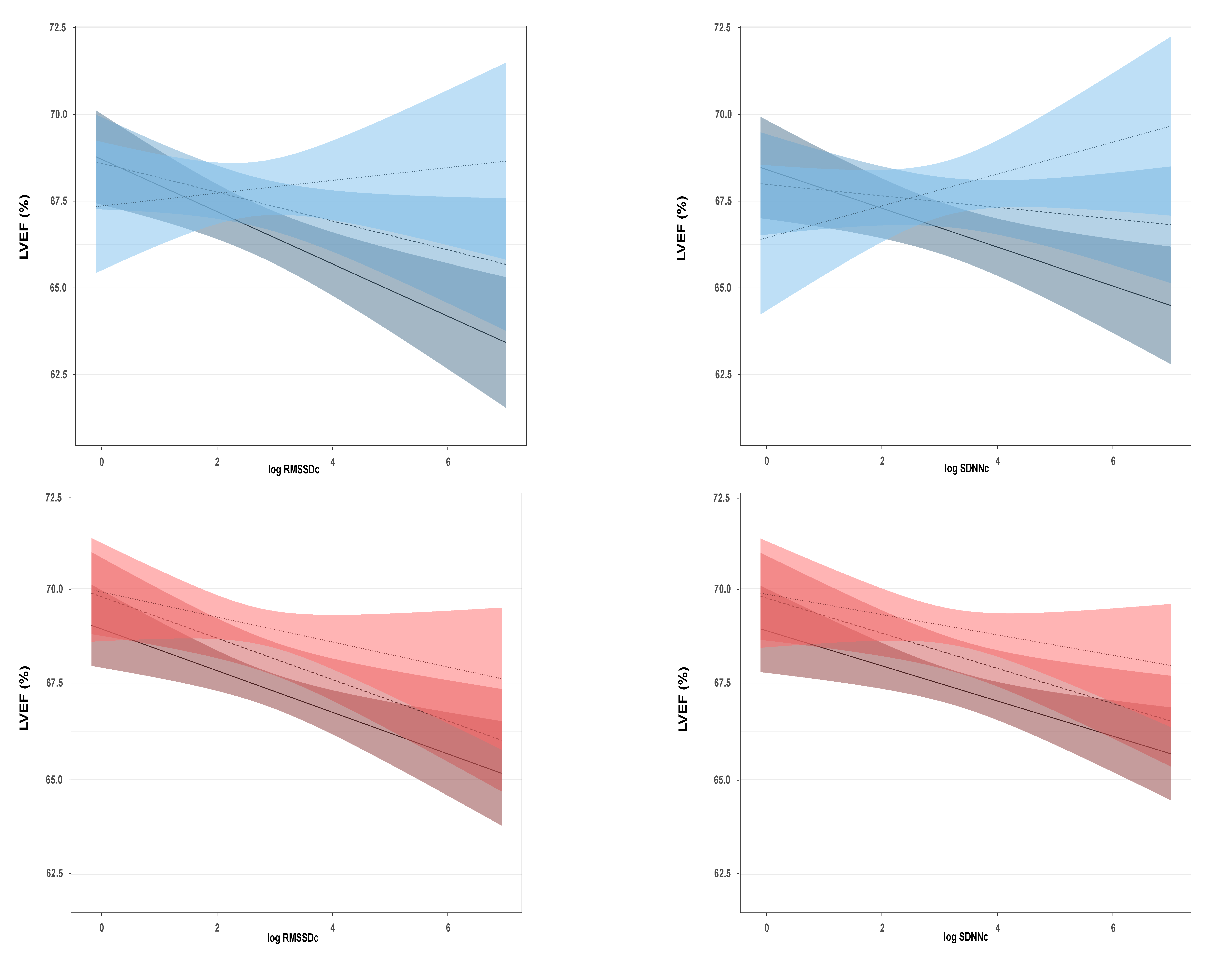


RMSSD: root mean square of successive RR-interval differences, SDNN: standard deviation of normal R-R intervals, LVEF: Left ventricular ejection fraction

**Figure S2.** Association of RMSSDc and SDNNc with E/A ratio at year 3, 6 and 9 of follow-up


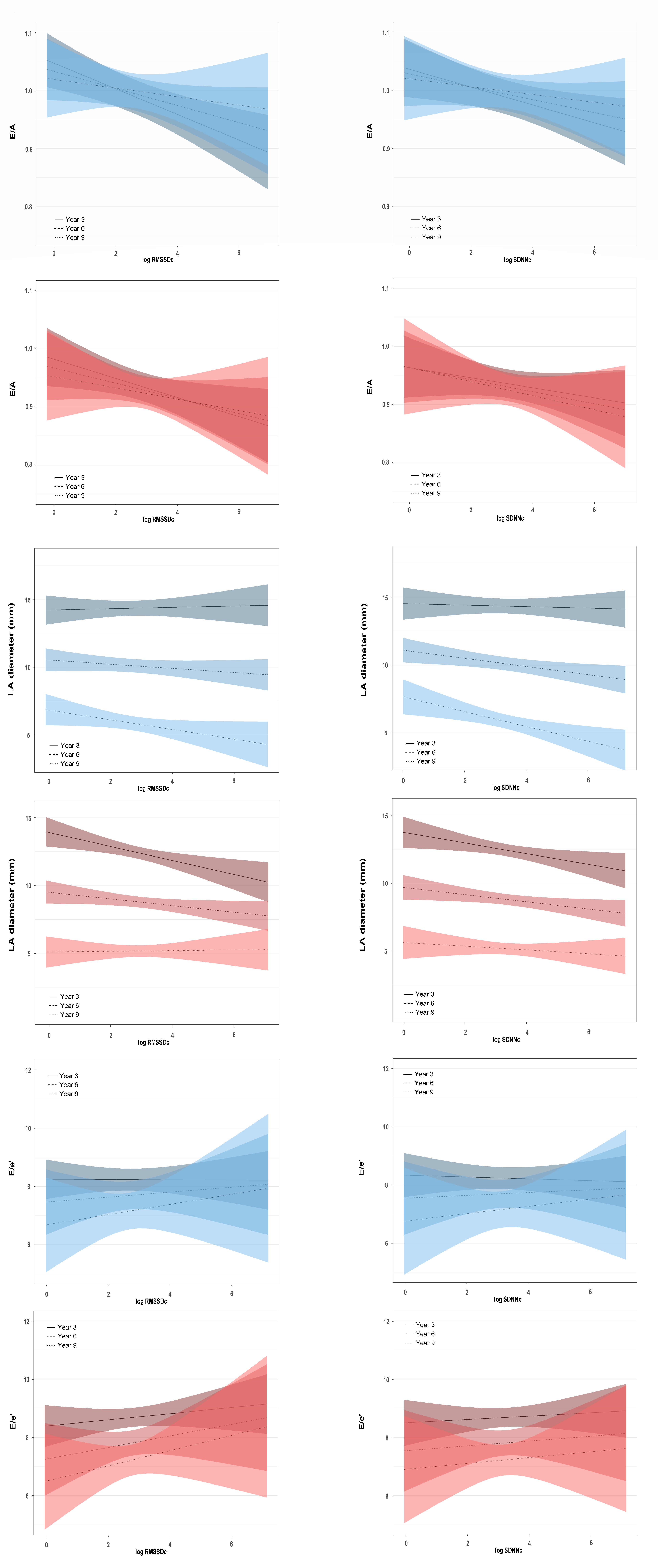


RMSSD: root mean square of successive RR-interval differences, SDNN: standard deviation of normal R-R intervals, E/A; mitral E wave/ A wave ratio. Analyses are adjusted for age, heart rate, hypertension, diabetes, CHD, total cholesterol and HDL, use of lipid lowering and cardiac medication, BMI.

**Figure S3.** Association of RMSSDc and SDNNc with LA diameter at year 3, 6 and 9 of follow-up


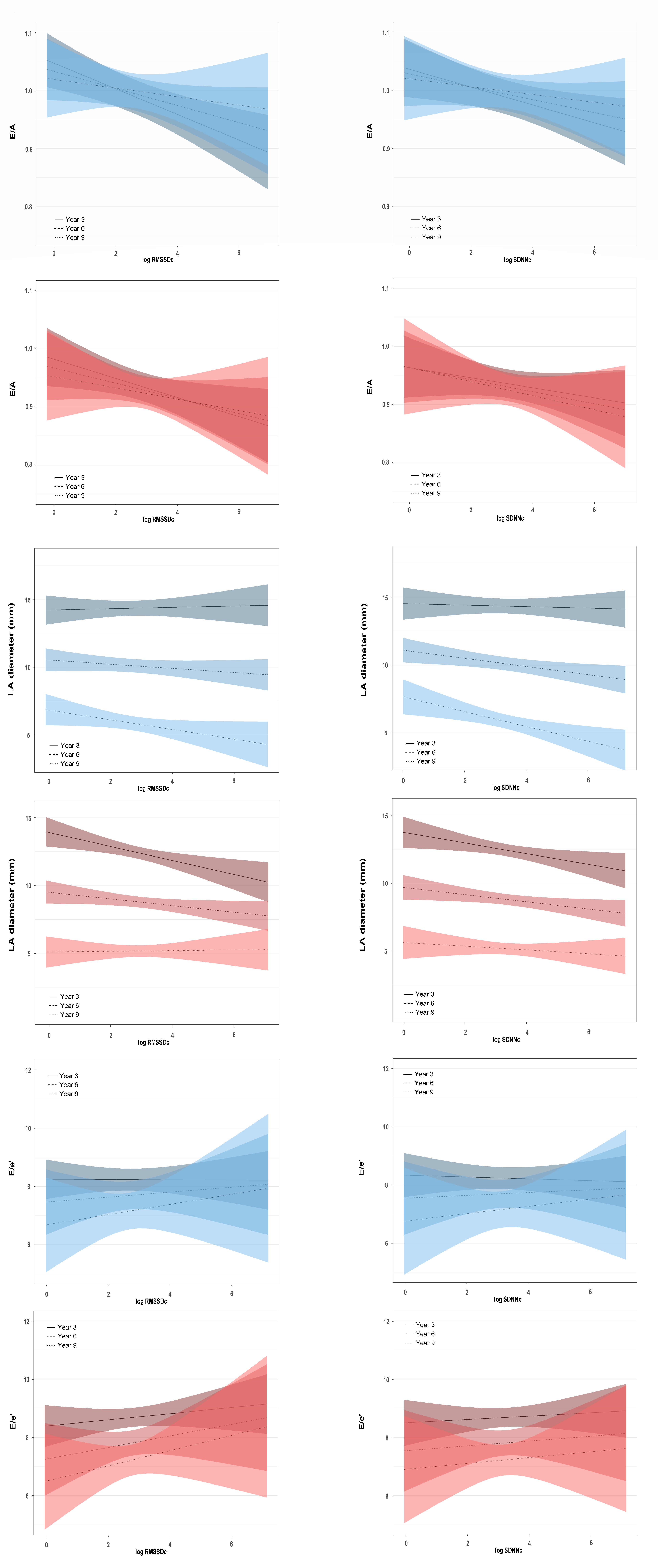


RMSSD: root mean square of successive RR-interval differences, SDNN: standard deviation of normal R-R intervals, LA diameter: left atrial diameter. Analyses are adjusted for age, heart rate, hypertension, diabetes, CHD, total cholesterol and HDL, use of lipid lowering and cardiac medication, BMI.

**Figure S4.** Association of RMSSDc and SDNNc with E/e’ ratio at year 3, 6 and 9 of follow-up


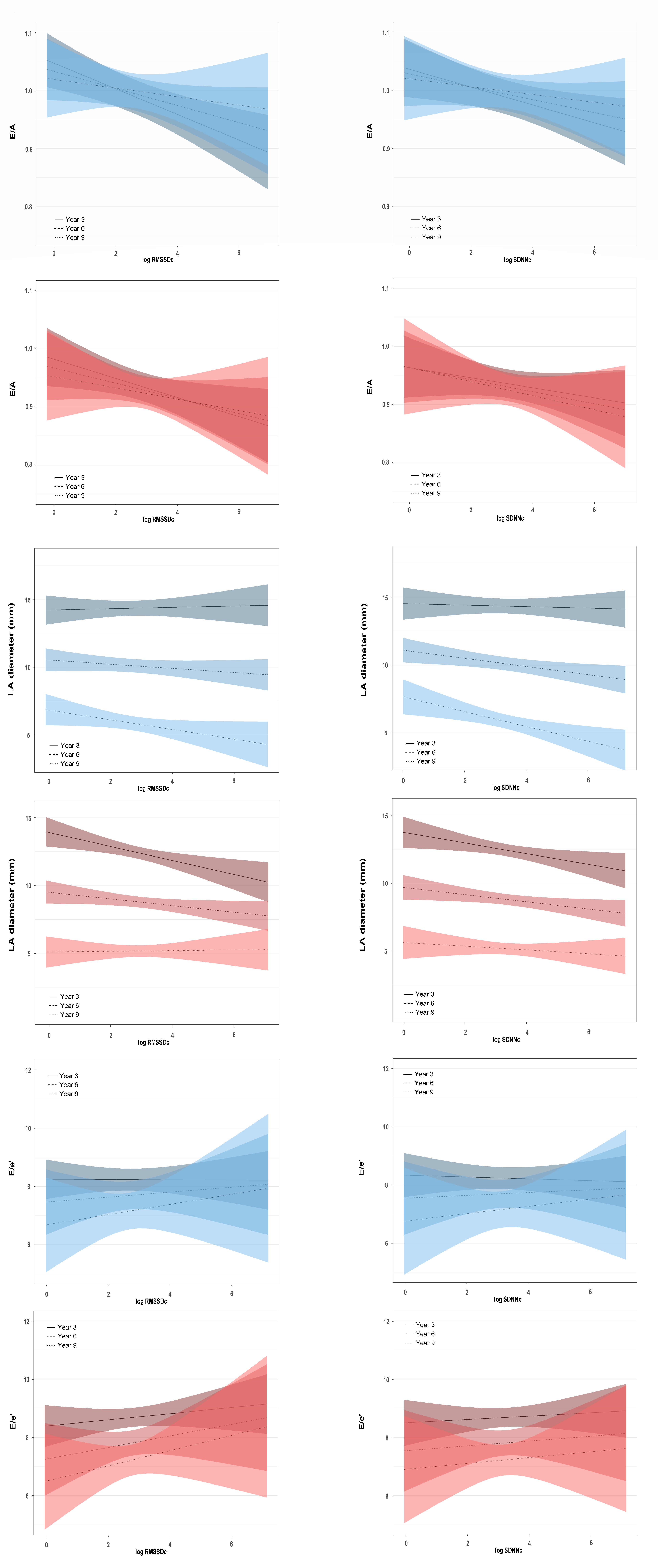


RMSSD: root mean square of successive RR-interval differences, SDNN: standard deviation of normal R-R intervals, E/e’: E wave/ septal e’ ratio. Analyses are adjusted for age, heart rate, hypertension, diabetes, CHD, total cholesterol and HDL, use of lipid lowering and cardiac medication, BMI.


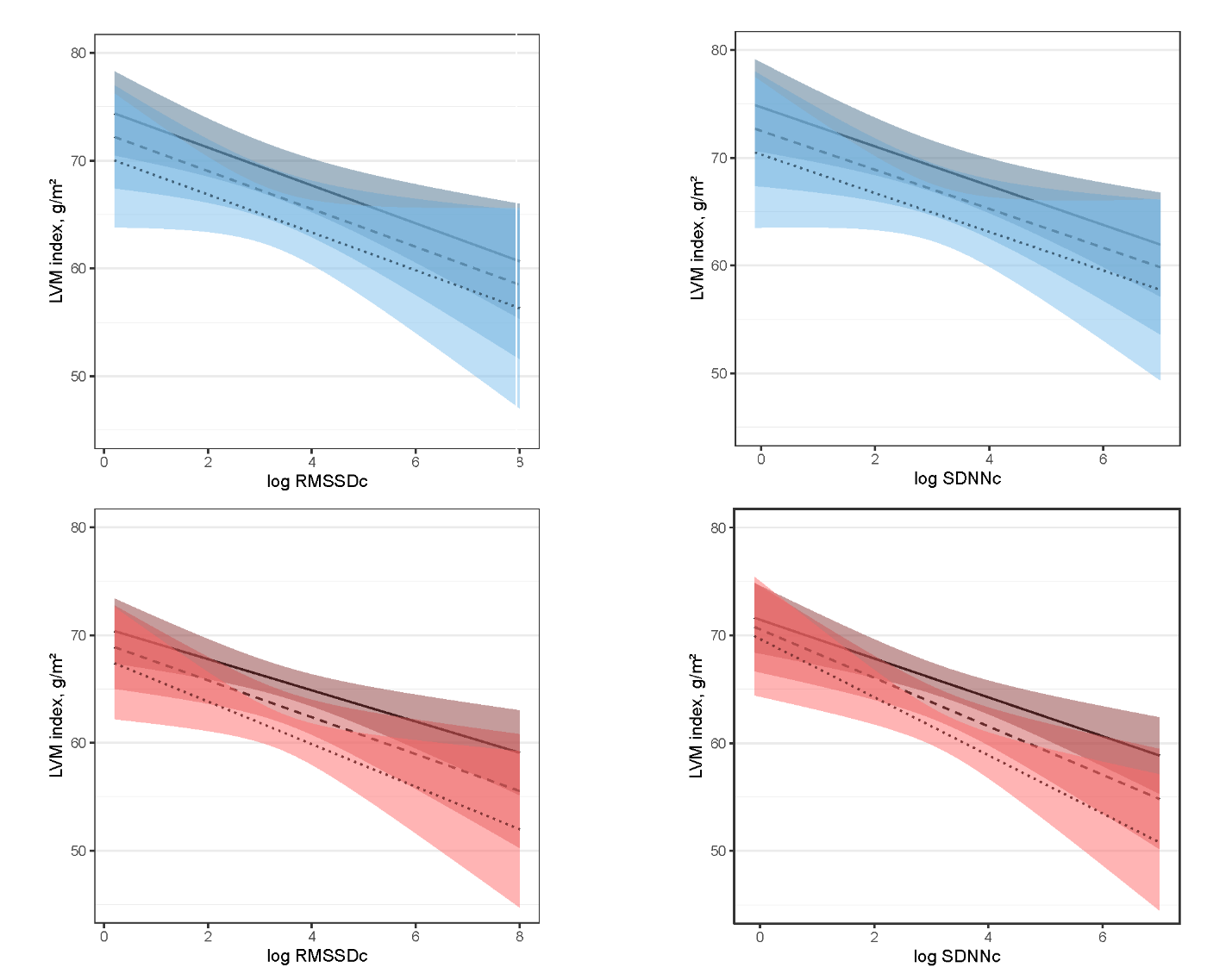
 **Figure S5.** Association of RMSSDc and SDNNc with LVM index at year 3, 6 and 9 of follow-up

RMSSD: root mean square of successive RR-interval differences, SDNN: standard deviation of normal R-R intervals, LA diameter: left atrial diameter. Analyses are adjusted for age, heart rate, hypertension, diabetes, CHD, total cholesterol and HDL, use of lipid lowering and cardiac medication, BMI.

**Methods S1.** Details on methods of data collection in the study

*Echocardiography*

Participants of the RS went through resting transthoracic two-dimensional, M-mode and Doppler echocardiography according to a standard protocol from RS-I-4, RS-II-2 and RS-III-1 onwards (number of individuals with echocardiography data were RSI-4 and RSII-2= 5301; RSIII-1=3644; RSI-5,RSII-3and RSIII-2= 6269; RSI-6 and RSII-4=1669). Echocardiographic examinations were performed by a commercially available ultrasonography system (AU3 Partner, Esaote Biomedica, with a 3.5/2.5 MHz transducer) until October 2003 and from then on by a commercially available Acuson Cypress, Siemens, USA with a 3V2c transducer. As of January 2009, a Vivid I (Vivid I, GE Healthcare, Little Chalfont, UK) with a 3S-RS Sector Array probe (1.5-3.6 MHz) was used. All examinations were performed by the same trained personnel, using the same protocol. At the time of study analysis, a maximum of three measurements were available for the study participants.

For LV systolic function, LVEF(%) was calculated based on the Teichholz formula. As for LV diastolic function parameters, early transmitral ventricular diastolic filling velocity (E wave) and late diastolic filling velocity (A wave) during three cardiac cycles were used to calculate E wave/A wave ratio (E/A) and left atrial anteroposterior diameter (LA diameter) was also measured. In a subset of 5182 participants form RS-I-5, RS-II-4 and RS-III-2, the early diastolic longitudinal filling velocity of the septal mitral annulus (septal e′) measurement was incorporated to calculate the E/e’ ratio (E/e’). Finally, we quantified LV mass indexed by body surface area (LVM index) using the cube formula.

*HF assessment*

Ascertainment HF diagnosis in the RS has been previously explained in detail. In short, prevalent HF at entry of the original Rotterdam study cohort was based on clinical information from medical records for all participants and by using a validated score, similar to the definition of HF by the European Society of Cardiology. In the subsequent cohorts, medical records of participants were screened for prevalent HF at entry. Thereafter, incidence of HF during follow-up was defined based on clinical information continuously collected from the medical records. Event date for HF was defined as the date of the first manifestation of symptoms suggestive of HF obtained from the medical records, from the day of receipt of a first prescription for a loop diuretic or an angiotensin-converting enzyme inhibitor, whichever came first.

Follow-up information for incident HF was complete until January 2016.

*Cardiovascular risk factors*

Data collection for the cardiovascular risk factors has been previously reported. Systolic and diastolic blood pressure were measured using at random-zero sphygmomanometer on the right arm. Two measurements were performed and the average of the two was used. Hypertension was defined as a systolic blood pressure ≥140 mm Hg, diastolic blood pressure ≥90 mm Hg or use of antihypertensive medication. The indication for use of antihypertensive medication was ascertained by a physician. Body mass index (BMI) was calculated based on weight in kilograms divided by height in meters squared (kg/m2). Serum total and high-density cholesterol (HDL) were measured using standard techniques. Smoking status (current, former and never), use of cardiac (digoxin, nitrates, and antiarrhythmic medication) and lipid lowering medication were extracted from questionnaires. Diabetes was defined as fasting blood glucose≥126 mg/dL (6.9 mmol/L) or use of glucose-lowering medication. Prevalent coronary heart disease (CHD) was defined as a history of myocardial infarction, coronary artery bypass grafting or percutaneous transluminal coronary angioplasty verified from medical records.
